# The Future of Gastrointestinal Physiology: 2020 and Beyond

**DOI:** 10.3389/fphys.2021.674951

**Published:** 2021-04-12

**Authors:** Stephen Pandol

**Affiliations:** Division of Gastroenterology, Cedars Sinai Medical Center, Los Angeles, CA, United States

**Keywords:** gastrointestinal, gastroenterology, gut, physiology, microbiome

## Gastrointestinal Sciences

There have been dramatic advances in gastrointestinal sciences over the past 10 years since the inauguration of the Frontiers in Gastrointestinal Sciences. In 2010, I suggested that the field would rapidly embrace the findings about the role of the gut microbiome in physiologic homeostasis as well as disorders of homeostasis; and that there would be a significant advance in our understanding of gut sensors and their connection to neurohumoral pathways mediating systemic responses (Pandol, 2010). Importantly, dietary nutrients and metabolic products of the dietary nutrients could play an important role in regulating systemic responses through the gut sensors. Frontiers has contributed importantly and broadly to advances in these areas and has provided a multidisciplinary platform for integrating the sciences involved. The specifics of cellular physiology and pathways are critical components to our mechanistic understanding of physiology at multiple organs and tissues, the functions of the gastrointestinal tract and its effects on systemic physiology are known to be regulated by what is going on in the gut lumen from dietary constituents ingested, the effects of ingested nutrients on the gut microbiome and the multiple pathways stimulated at the gut wall that have profound and widespread effects on the entire body. Thus, what happens in the lumen of the gastrointestinal tract can have broad effects in homeostasis and disease.

Regarding the gut microbiome, I thought that interdisciplinary scientific approaches would start to reveal the interplays between diet, the gut microbiome epithelial function and immune functions, and systemic metabolism. Disorders of one or more participants in this interplay would likely underlie some of the most common and devastating medical problems of our time including the metabolic syndrome (obesity, diabetes mellitus, hypertension and liver, and vascular diseases), inflammatory gastrointestinal diseases and predisposition to several cancers. This call was followed by an inspirational review Frontiers published by Grenham et al. (2011) providing observations of the role of the gut microbiome in intestinal function including proliferation and differentiation of the epithelium and the development of the immune system and activation of neural pathways. Grenham et al. (2011) also outlined hypotheses about the role of the gut microbiome and the central nervous system (CNS) indicating that CNS stress may cause gut disorders of such as irritable bowel syndrome through effects on the gut microbiome; and that disorders in the gut microbiome could have effects on the CNS. Another highly cited review by Greer and O'Keefe (2011) reviewed evidence suggesting that the westernized diet, the overuse of antibiotics and decreased breast feeding through alterations in the gut microbiota promotes the inflammatory potential in the bowel creating increased risk for inflammatory diseases and cancer of the bowel in those with a genetic predisposition. The review further points out the increased risk of these disease is associated with the suppression of microbial fermentation and production of butyrate which is anti-inflammatory and anti-proliferative (Greer and O'Keefe, 2011).

More recently, Bliss and Whiteside (2018) provided a framework for understand the complex relationship between the Western diet, the gut microbiome, lumenal sensors on gut enteroendocrine cells axis via local, paracrine and/or endocrine mechanisms involving a variety of gut-derived peptides produced from enteroendocrine cells. The sensors on the strategically located gut enteroendocrine cells interacting with meal nutrients and microbial metabolites activate intracellular signaling systems to release hormones including cholecystokinin (CCK), glucagon-like peptide 1 (GLP-1), and peptide YY (PYY). We know that these hormones have regulator effects on a large number gastrointestinal organs as well as the CNS. Some of these effects include slowing of gastric emptying, secretion of biliary and pancreatic enzymes, promotion of insulin secretion and inhibition of feeding. Not only do CCK, GLP-1, and PYY have hormonal effects but they have paracrine effects on neural sensory pathways carrying information in the enteric nervous system to the CNS and then to motor pathways having further regulatory effects. Finally, recent findings show that the enteroendocrine cells have neural properties and directly synapse with the neurons of the enteric nervous system (Liddle, 2019). In combination, these findings indicate that nutrients, and microbial metabolic products not only have local effects on epithelial function but have widespread effects systemically through the immune system, hormone secretion and neural pathways that connect to the CNS.

Since these inaugural articles there has an avalanche of scientific papers on the gut microbiome in the literature. A PubMed search shows that in 2010 there were <100 papers with the topic of gut microbiome while in 2020 the number was just under 7,000- a near 700 times increase. Frontiers in Gastrointestinal Sciences has played a significant role in publishing important papers in the emerging field. These include papers on the role of the gut microbiome in both alcoholic (Su et al., 2016; Yue et al., 2020; Zheng et al., 2020) and non-alcoholic (Bian et al., 2017; Porras et al., 2018; Zheng et al., 2018) liver disease; the role of sulfur metabolism by the microbiome on gut sensory signaling, colonic inflammation and cancer; (Carbonero et al., 2012; Bala et al., 2014; Guo et al., 2016; Burgueño et al., 2019; Fei et al., 2019; Song et al., 2019; Ren et al., 2020) glucose homeostasis and type 2 diabetes; (Lê et al., 2012; Su et al., 2016) tuberculosis (Luo et al., 2017) mood and behavior and Gulf war illness; (Grenham et al., 2011; Kimono et al., 2019) pulmonary tuberculosis; (Luo et al., 2017) and autoimmune pancreatitis (Haruta et al., 2012). Other papers provided information on the effects of xenobiotics, probiotics, vitamins, and medication affect the gut microbiome and physiology (Yang et al., 2015; Su et al., 2016; Atashgahi et al., 2018; Li et al., 2018; Zheng et al., 2018; Chang et al., 2019; Fei et al., 2019; Guan et al., 2019; Song et al., 2019; Xu et al., 2019; Zhu et al., 2020) while some addressed the interplay between endogenous processes and the gut microbiome including bile acids and cholesterol metabolism, adrenergic input and gut epithelial responses (Yang et al., 2017; Burgueño et al., 2019; Molinero et al., 2019).

This discussion is not meant to suggest that important observations in gastrointestinal sciences outside of the microbiome are not highly considered by Frontiers. On the contrary, the discussion is meant to suggest that the expansion of the field will occur with greater mechanistic understanding of impact of the microenvironment of the gut organs including dietary constituents, xenobiotics and metabolic products of the microbiome have on the secretory, absorptive, elimination, motility, endocrine, neural and immune functions of the gastrointestinal system. As the field progresses greater use of co-culture of *in vitro* and *ex vivo* models including organ on a chip technology with microorganisms; (Pearce et al., 2018) and use of germ-free animal models (Kennedy et al., 2018) to selectively introduce a microbiome under investigation will be encouraged. This article is meant to encourage continued multidisciplinary approaches to further the progress in gastrointestinal sciences so that new understanding can be translated to benefit human health.

## Author Contributions

The author confirms being the sole contributor of this work and has approved it for publication.

## Conflict of Interest

The author declares that the research was conducted in the absence of any commercial or financial relationships that could be construed as a potential conflict of interest.
