# Corrigendum: The Future of Gastrointestinal Physiology: 2020 and Beyond

**DOI:** 10.3389/fphys.2021.776954

**Published:** 2021-09-29

**Authors:** Stephen Pandol

**Affiliations:** Division of Gastroenterology, Cedars Sinai Medical Center, Los Angeles, CA, United States

**Keywords:** gastrointestinal, gastroenterology, gut, physiology, microbiome

The title in the originally published article was incorrect. Instead of “The Future of Physiology: 2020 and Beyond” it should be “The Future of Gastrointestinal Physiology: 2020 and Beyond.”

The authors apologize for this error and state that this does not change the scientific conclusions of the article in any way. The original article has been updated.

## Publisher's Note

All claims expressed in this article are solely those of the authors and do not necessarily represent those of their affiliated organizations, or those of the publisher, the editors and the reviewers. Any product that may be evaluated in this article, or claim that may be made by its manufacturer, is not guaranteed or endorsed by the publisher.

